# Europium-Doped Tellurite Glasses: The Eu^2+^ Emission in Tellurite, Adjusting Eu^2+^ and Eu^3+^ Emissions toward White Light Emission

**DOI:** 10.3390/ma12244140

**Published:** 2019-12-10

**Authors:** Hagar Elkholy, Hosam Othman, Ibrahim Hager, Medhat Ibrahim, Dominique de Ligny

**Affiliations:** 1 Physics Department, Faculty of Science, Menoufia University, Shebin El-Kom 32511, Egypt; hosamssl4@yahoo.com (H.O.); izhager@yahoo.com (I.H.); 2 Department Werkstoffwissenschaften, Lehrstuhl für Glas und Keramik, Universität Erlangen-Nürnberg, Martensstrasse5, D-91058 Erlangen, Germany; dominique.de.ligny@fau.de; 3 Spectroscopy Department, National Research Centre, El-Buhooth Str., Dokki 12622, Giza, Egypt; medahmed6@yahoo.com

**Keywords:** tellurite glass, Eu^2+^ and Eu^3+^emission, ATR, emission decay kinetics

## Abstract

Europium-doped magnesium tellurite glasses were prepared using melt quenching techniques and attenuated total reflection (ATR) spectroscopy was used to study the glass structure. The glass transition temperature increased with increasing MgO content. Eu^2+^ and Eu^3+^ emissions were studied using photoluminescence spectroscopy (PL). The broad emission of Eu^2+^ ions centered at approximately 485 nm was found to decrease in intensity with increasing MgO content, while the Eu^3+^ emission was enhanced. The Eu^3+^ emission lay within the red orange range and its decay time was found to increase with increasing MgO content. Different excitation wavelengths were used to adjust Eu^2+^ to Eu^3+^ emissions to reach white light emission. The white light emission was obtained for the sample with the lowest MgO content under excitation in the near-UV range.

## 1. Introduction

Tellurite glasses are known for their low phonon energy, ≤800 cm^−1^, much lower than borate, phosphate, and silicate glasses. Beside their high liner refractive index (≈2), their nonlinear refractive index is also high, making tellurite glasses excellent candidates for use in second- and third-generation harmonic applications [1,2]. Their overall high optical basicity (^th) will enhance the reduction of doped elements. Tellurite glasses are also known for their low glass transition temperature (T_g_) and low melting (T_m_) temperatures. They can be doped with high concentrations of rare-earth ions (REs), 50 times higher than silicate glasses, without presenting any quenching effect [3,4].

Doping glass with REs ions is of great interest due to the number of application for which these glasses could be suitable, such as optical fibers, color displays, waveguides, solar cells, emitting diodes, optical switches, lasers, optical detectors, and optical amplifiers [5,6,7]. The f–f transitions of RE ions are not highly influenced by the change in the ligand field due to the high shielding. Where the hypersensitive transitions are electric dipole ones, such as in Sm^3+^ (^6^H_5/2_→^6^F_1/2_), Dy^3+^ (^6^F_11/2_→^6^H_15/2_), and Eu^3+^ (^5^D_0_→^7^F_2_), they are highly influenced by any changes in the host [8,9,10]. Two stable oxidation states of Europium were observed in glasses, namely, divalent and trivalent oxidation states [11,12,13]. The divalent state Eu^2+^ of Europium is characterized by a broad emission band extending from the ultraviolet to blue range, that is 4f^6^5d→4f^7^, a partially allowed transition which is sensitive to any structural changes in the surrounding host [11,14]. The Eu^3+^ emission is attributed to f–f transitions, which are not highly affected by the crystal field. Nevertheless, the hypersensitive transition ≈614 nm that is an electric dipole transition is influenced by the ionicity and symmetry of the host, while the pure magnetic dipole transition ≈592 nm is less sensitive to the environment. Hence, the ratio of emission of hypersensitive transition to that of the pure magnetic is used as a probe for structural changes [15]. White light emission could be obtained from the combination of the emission from Eu^2+^ and emission of the Eu^3+^ [16,17] in silicate and phosphate glasses. However, Eu^3+^ is difficult to reduce and needs special treatment such as melting in a graphite furnace or nitridation.

Associating the high optical basicity of Tellurite glasses with Europium doping should lead to a high content of Eu^2+^ and then a suitable white light emitter. In this paper, we doped a TeO_2_–MgO binary series showing variations of TeO_4_ and TeO_3_ structural units of tellurite. The structural modifications of the doped glasses were studied by Infrared spectroscopy. To the best of our knowledge, the emission of Eu^2+^ has rarely been investigated in tellurite glass. In this paper, we studied Eu^2+^ emission in the prepared glass without any reducing conditions. The effect of glass composition and structure on the ratio of blue emission from Eu^2+^ and red emission from Eu^3+^ was studied. These results allowed us to optimize a promising white light emitter.

## 2. Materials and Methods

Magnesium tellurite glass containing europium, of the general form (99-x)TeO_2_-xMgO-1Eu_2_O_3_, where x = 9, 19.29, and 39 mol%, was prepared using a melt quenching technique. This sample was designated TMgxEu1. High purity dried chemicals (TeO_2_ (Alfa Asser 99.99%) and MgO (Sigma-Aldrich 99.99%)) and Eu_2_O_3_ (Sigma-Aldrich 99.9999%)) were weighed, mixed together using an agate mortar, and transferred in a Pt crucible to a furnace at melting temperature, varying between 850 and 1100 °C, for approximately 40 min. The melted mixture was stirred several times during the melting process. The molten mass was cast in a stainless steel mold (≈150 °C), and then transferred immediately to the annealing furnace. The annealing temperature varied between 300 and 420 °C, depending on the composition, for approximately 2 h. The results presented here were compared to our previously synthesized Eu-free glass series, (100-x)TeO_2_-xMgO, designated TMgx. This reference series was synthesized and annealed under the same conditions.

X-ray diffraction (XRD) measurements were done using a Brucker D8 advance eco X-ray diffractometer, with λ = 1.54178 Å, a power source of 40 KeV and 25 mA, and a graphite monochromator for Cu-Kα radiation.

The glass transition temperature, T_g_, of the prepared glasses was obtained through differential scanning calorimetry (DSC), (NETZSCH DSC 404F1), using a constant heating rate of 10 °C min^−1^ with an error of ±1.5 °C. T_g_ was determined as the onset temperature.

The attenuated total reflection (ATR) spectra were collected using Thermo Nicolet iS 10, (ThermoFisher Scientific, Germany) using Diamond crystal with *n* = 2.4 from Czitek.

The photoluminescence spectra were measured using a spectrofluorometer equipped with double monochromators (Czerny-Turner) in excitation and emission (Fluorolog3, Horiba JobinYvon), using a 450 W Xe-lamp as the excitation source (the excitation and emission spectral resolution was 1 nm). The decay spectra were collected using the flouroHub and data station software.

## 3. Results and Discussion

### 3.1. Structural and Thermal Investigation

**Figure 1:**
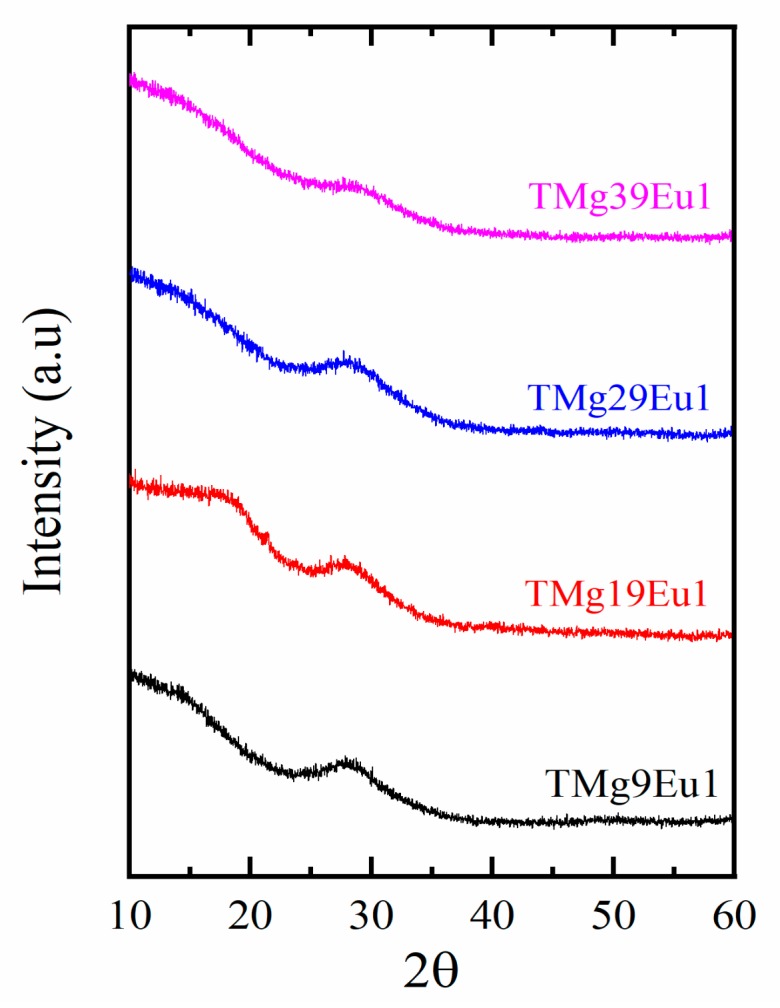
XRD spectra of the prepared (xTeO_2_-(99-x)MgO-1Eu_2_O_3_) glasses.

Since the 1 mol% of Eu_2_O_3_ doping is a high concentration, the risk of crystallization is a concern. The amorphous nature of the prepared Europium-doped magnesium tellurite glasses was confirmed by XRD measurement. The results are shown in Figure 1. No sharp crystalline peaks are observed, only the hump around 30° that is characteristic for glasses.

**Figure 2:**
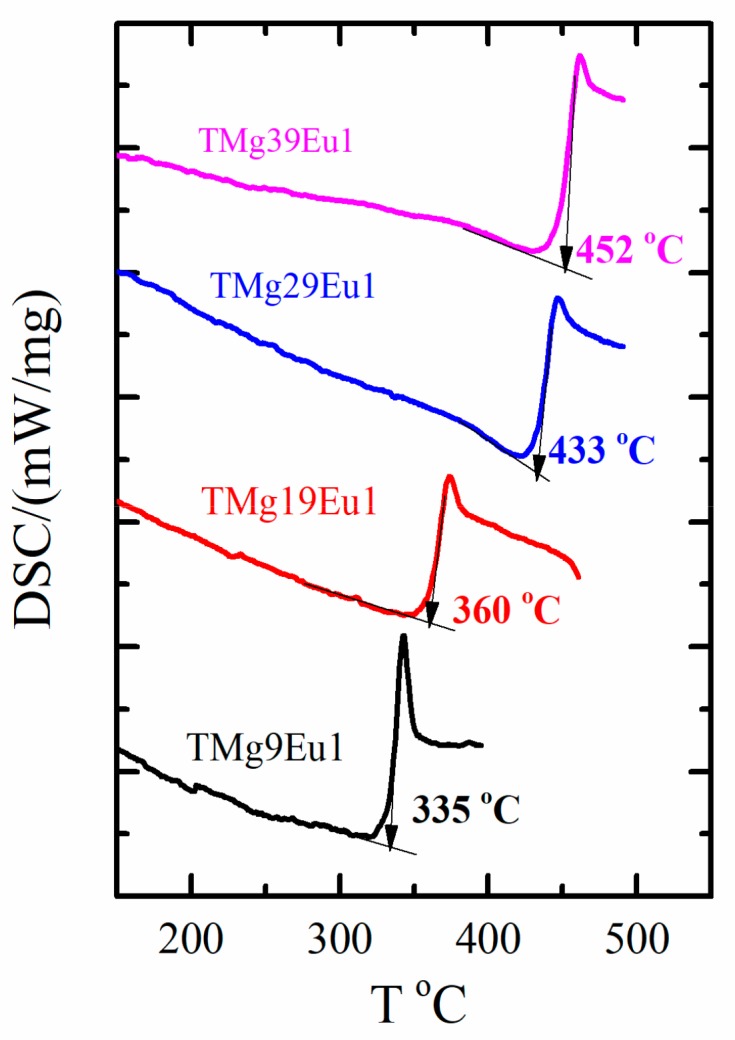
Differential scanning calorimetry (DSC) spectra for T_g_ of (xTeO_2_-(99-x)MgO-1Eu_2_O_3_) glasses.

The glass transition temperature (T_g_) curves for the prepared glasses are represented in Figure 2.

Here, an increase in the T_g_ can be observed when the MgO content is increased. However, when compared with the undoped glasses [18], the T_g_ is found to be higher for the doped glasses than that of the undoped glasses reported in our previous work (see Table 1). This behavior is attributed to the higher bond enthalpy of Eu-O (557 KJ/mol) compared to that of Mg-O (358 KJ/mol) [19,20].

**Figure 3:**
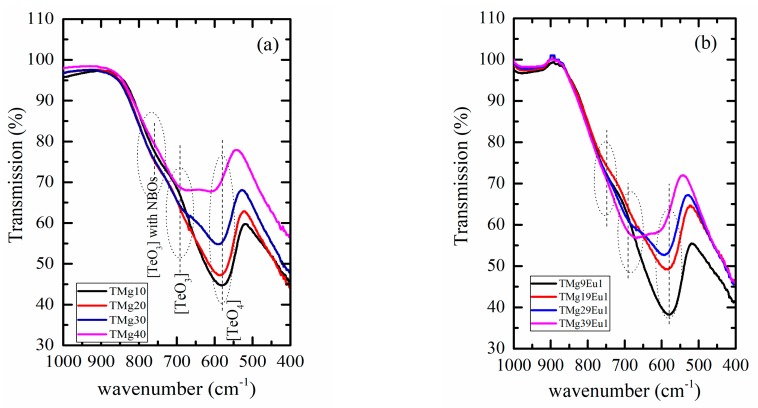
Attenuation of Total Reflectance (ATR) transmission spectra: (**a**) for the (TMgx) undoped series; (**b**) for the (TMgxEu1) doped series. The peak position of the TeO_3_ with the non-bridging oxygen (NBOs) line was fixed at 760 cm^−1^, the TeO_3_ at 700 cm^−1^, and TeO_4_ at 580 cm^−1^.

Incorporation of oxide modifier into the tellurite network leads to the change of TeO_4_ trigonal bipyramid to polyhedral TeO_3+1_ and trigonal pyramid TeO_3_ structural units, and is associated with the formation of non-bridging oxygen (NBOs) [21]. Figure 3 shows the ATR spectra of undoped (TMgx) and (TMgxEu1) doped series.

The spectra are dominated by a broad band extending from 500 to 900 cm^−1^. Although the ATR method is preferred over the KBr technique as it eliminates the effect of varying glass content in the KBr powder, the absolute intensity could be affected by the degree of contact between the glass powder and the diamond crystal. Hence, to overcome this problem, we used the normalized spectra and compared the relative rather than the absolute intensities. First, the peak centered at 590 cm^−1^, assigned to the [TeO_4_] trigonal bipyramid structural unit [22,23,24], shifts toward higher energies from 580 to 615 cm^−1^ with increasing MgO content. This variation is similar for both the doped and undoped series, along with the decrease in its intensity. The absorption band centered at approximately 700 cm^−1^ is assigned to the stretching vibrations of the trigonal pyramid (TeO_3_) units [24,25,26] and it shows a slight shift toward lower energies (from 672 to 665 cm^−1^) with increasing MgO content. The relative contribution between the TeO_3_ and TeO_4_ units increases with increasing MgO and is more pronounced for the doped samples. This behavior confirms that, As MgO increases, the TeO_4_ decreases as it is converted to (TeO_3_) structural units and that Eu doping also contributes to this conversion. The last band centered around 770 cm^−1^ shows a slight shift to higher energies as MgO increases for both series. This center is attributed to the stretching vibration between tellurium and NBOs of the trigonal pyramidal (TeO_3_) structural units [27,28].

**Figure 4:**
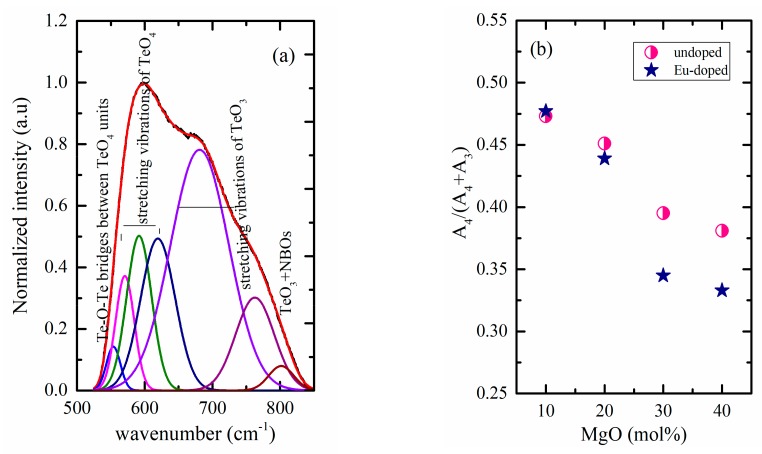
This figure shows: (**a**) An example of the deconvoluted ATR spectra; (**b**) the change in the area of the envelope related to the TeO_4_ (A_4_) to the total area (A_4_ + A_3_).

To have a better view of the structural changes, the spectra were converted to absorption. A baseline anchored at 525 and 850 cm^−1^ was subtracted and the spectra were normalized, and then deconvoluted using Gaussian shape components [29,30]. Figure 4a represents an example of the deconvoluted spectra. From the deconvoluted data, the change in the area of the bands related to the TeO_4_ (assigned as A_4_) and TeO_3_ (assigned as A_3_) and the ratio between areas of bands related to the TeO_4_ structural units to the total area was calculated and is plotted in Figure 4b. This ratio can be considered in a first approximation to be proportional to the concentration of the species and confirms the conversion of TeO_4_ to TeO_3_ units. The difference between undoped and doped glasses becomes greater the higher the MgO content (x = 29 and 39 mol%).

### 3.2. Photoluminescence Spectroscopy

#### 3.2.1. Photoluminescence of Eu^3+^

**Figure 5:**
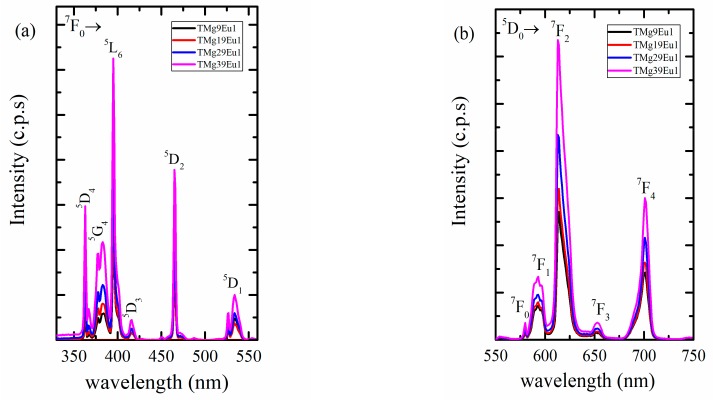
(**a**) Excitation spectra of Eu^3+^ at λ_em_ = 614 nm; (**b**) Emission spectra for Eu^3+^ at λ_ex_ = 392 nm for the prepared glasses.

The emission and excitation spectra for Eu^3+^ are presented in Figure 5. The excitation spectrum (Figure 5a) shows six lines, all originating from the ground state ^7^F_0_ to different excited states centred around 363, 380, 394, 416, 466, and 530 nm corresponding to the energy states ^5^D_4_, ^5^G_4_, ^5^L_6_, ^5^D_2_, and ^5^F_1_, respectively [31,32,33,34]. In all of the prepared glasses, the excitation spectra were identical, as expected since the f–f transitions were shielded. The maximum excitation at 392 nm, corresponding to the transition ^7^F_0_→^5^L_6_, was chosen to discern the emission spectra of Eu^3+^ ions presented in Figure 5b.

**Figure 6:**
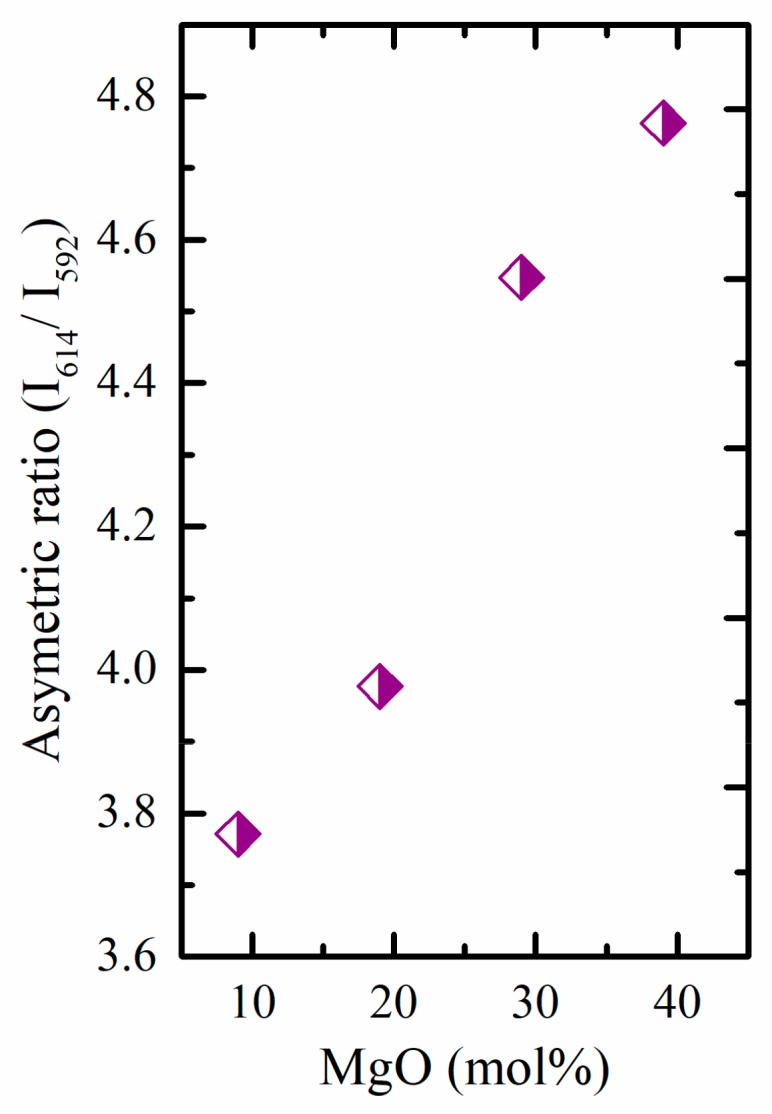
Graphic of the asymmetry ratio (I_614_/I_592_) of the prepared glasses.

The spectra are dominated by the characteristic emission lines of Eu^3+^ ions, all originating from the ^5^D_0_ to different lower energy states at approximately 592, 614, 653, and 701 nm, corresponding to the energy states ^7^F_0_, ^7^F_1_, ^7^F_2_, ^7^F_3_, and ^7^F_4_, respectively [31,34,35,36]. Here again, the emission spectra do not show any shift with composition; however, variations in intensities can be observed. The total intensity increases with the MgO content. The intensity ratio (I_614_/I_592_) is known as the asymmetry ratio and was used as indication of structural changes [34,37]. The increase in the intensity of the hypersensitive transition (^5^D_0_→^7^F_2_) assisted by the increase observed for the asymmetry ratio indicates that the Eu-symmetry site decreases with increasing the MgO content (see Figure 6). The asymmetric ratio is compared to other Eu-doped glasses in Table 2. These structural modifications around Eu ions can be correlated to the increase in the proportion of TeO_3_ units (Figure 4b). The increase in TeO_3_ units is combined with an increase in non-bridging oxygen, which could also affect the Eu^3+^ environment.

**Figure 7:**
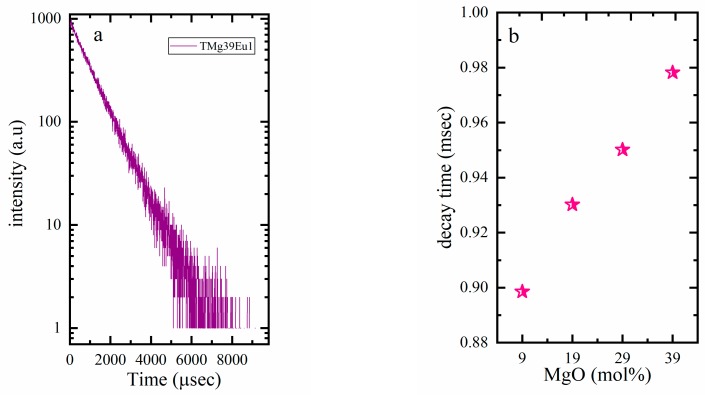
Graphic of (**a**) an example of the decay spectra; (**b**) the decay time of the prepared glasses.

Figure 7a shows an example of the decay curves. The decay is found to have a single exponential and this behaviour suggests that the Eu^3+^ is homogeneously distributed in the glass host. The decay times (τ) were obtained and used to calculate the decay rate (decay rate = 1/τ (ms^−1^)) (see Figure 7b).

The enhancement of the intensity of the hypersensitive transition ^5^D_0_→^7^F_2_ (Figure 5b) and the lower decay rate (Table 1) are understood in terms of the low phonon energy of tellurite, as well as the decrease in the optical basicity of the glass host, as these lead to more ionicity around Eu ions. The decrease in the optical basicity (see, Table 1) of the glass arises not only from the lower basicity of MgO (0.69), which replaces TeO_2_ (0.95), but is also due to the lower basicity of the TeO_3_ units that formed upon increasing MgO, than that of TeO_4_ units. Optical basicity was calculated theoretically according to the following relation [45,46]:

(1) ^th=XMgO^MgO+XTeO2^TeO2+XEu2O3^Eu2O3

where X_MgO_, X_TeO_2__, and X_Eu_2_O_3__ are the oxygen equivalent fraction and ^MgO and ^TeO2 are the optical basicity values of each oxide. ^MgO, ^TeO_2_, and ^Eu_2_O_3_ were taken as 0.69, 0.95, and 1.10, respectively [47,48].

#### 3.2.2. Toward White Light Emission

**Figure 8:**
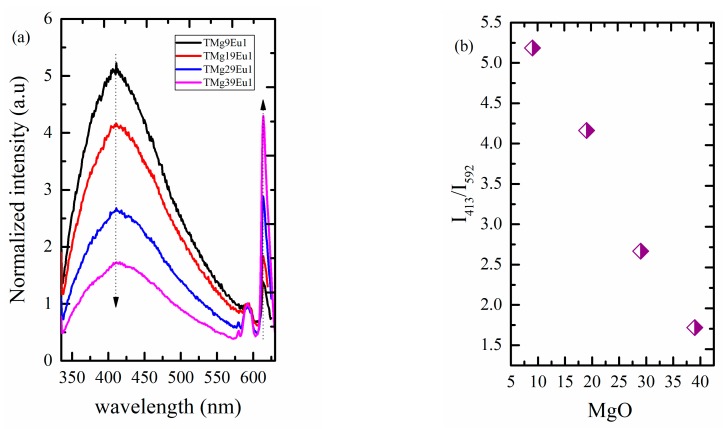
Graphics of (**a**) normalized emission spectra for the prepared glasses under λ_ex_ = 330 nm; (**b**) the ratio between the Eu^2+^ to Eu^3+^ emission at 413 nm of Eu^2+^and emission at 592 nm of Eu^3+^.

Divalent europium Eu^2+^ has a broad emission due to the 4f^6^5d→4f^7^ transition. This emission lies in the blue range and is strongly affected by any change in the ligand field of the host [35]. Figure 8 shows the emission spectra of Eu^2+^ ions for the prepared TMgxEu1 glass series excited at 330 nm. The emission of Eu^2+^ is almost constant and centered at ≈413 nm when compared with data reported for alkali phosphate excited using the same excitation wavelength [49], it was found to be lower in our case. A relationship between optical basicity and Eu^2+^ emission was proposed for phosphate, silicate, and borate glasses [35,49,50]; however, the current results show that tellurite glasses do not follow this relationship. Indeed, with an optical basicity almost double compared to silicates and phosphate, the Eu^2+^ emission in tellurite is at almost the same position.

Looking now at the intensity, it can be seen that the Eu^3+^ emission was enhanced compared to Eu^2+^ emission with increasing MgO content (Figure 8).

**Figure 9:**
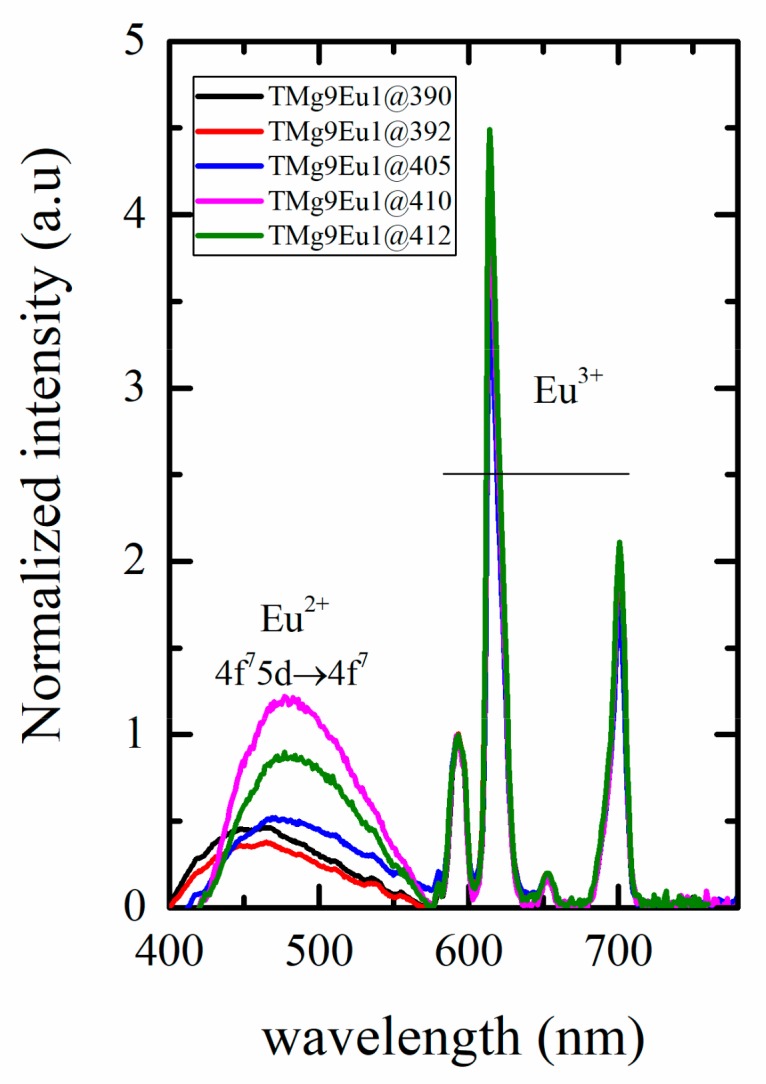
Normalized emission spectra of the TMg9Eu1 glass sample under different excitation wavelengths. The normalization is done to the pure magnetic transition of Eu^3+^ at 592 nm.

Figure 8b shows the ratio between Eu^2+^ and Eu^3+^ emissions at 413 nm and 592 nm, respectively. The emission at 592 nm was chosen because it is a pure magnetic transition that is not affected by any structural change around the RE ions. It can be used as a reference and its intensity is proportional to the Eu^3+^ concentration. This ratio decreases with increasing MgO content, which can be understood as being due to a change of the redox state of Eu or to a decrease of Eu^2+^ emission. It is known that the intensity and the position of Eu^2+^ can be affected by the ligand field, doping concentration, and by the excitation wavelength; therefore, we first discuss how it would be affected by the change in the excitation wavelength. Figure 9 shows an example of how the Eu^2+^ emission changes under different excitation wavelengths for the glass sample TMg9Eu1. The emission of Eu^2+^ was recorded along with that of Eu^3+^ over the range 430–780 nm under different excitation wavelengths. A shift toward a lower energy could be observed upon increasing the excitation wavelength. The emission shifts from 455 nm to 483 nm between an excitation at 390 and 412 nm.

**Figure 10:**
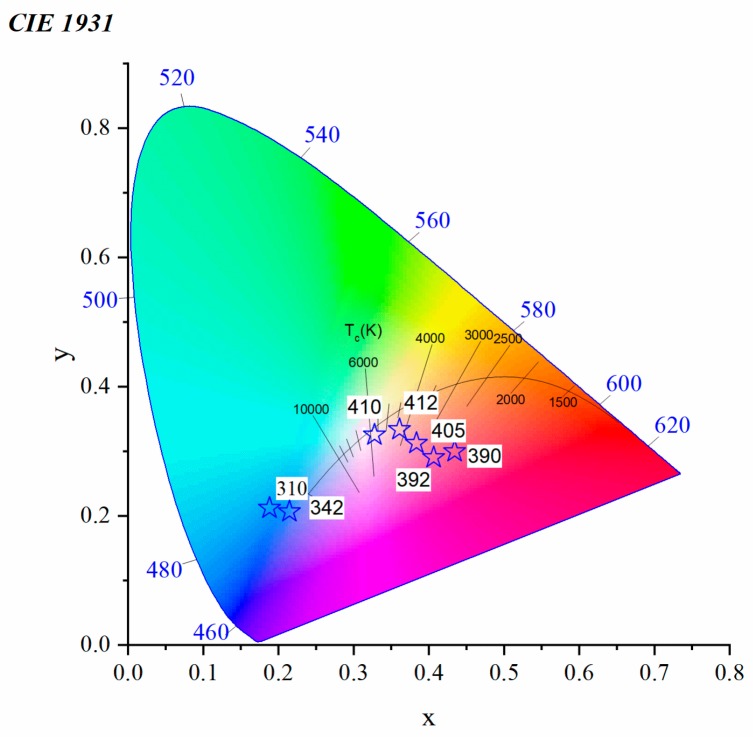
Commission International de I’Eclairage (CIE) 1931 chromaticity diagram of a TMg9Eu1 glass sample under different excitation wavelengths.

The Eu^2+^ emission intensity increases when the excitation is changed from 390 to 410 nm and then decreases with higher excitation, here 412 nm. The shift of the Eu^2+^ emission alone has a very small effect on the colour. However, the variation of the intensity ratio between the blue Eu^2+^ and the red Eu^3+^ provides us with a good chance to maintain white emission by tuning the excitation wavelength. From the spectra of Figure 9, the colour coordinate associated with each excitation can be computed as shown in Figure 10.

**Figure 11:**
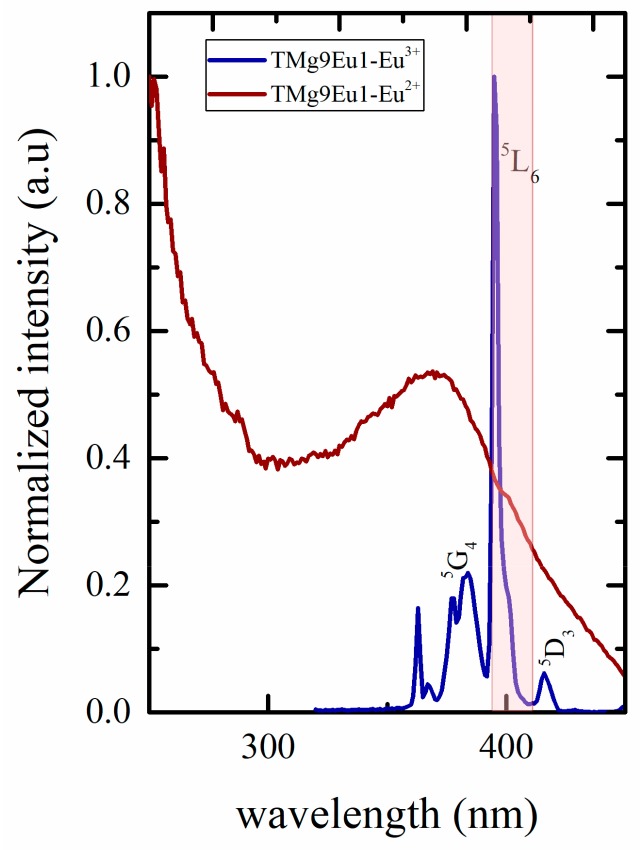
Superposition of the normalized excitation spectra of Eu^2+^ and Eu^3+^; shaded region represents the tuning excitation region that was used to reach the white color.

A low excitation wavelength (310, 342 nm) is out of any excitation lines for Eu^3+^ and the colour emitted is dominated by the blue Eu^2+^ emission. To understand better these variations of colours, excitation spectra for both ions are plotted in Figure 11, where the excitations from 392 to 412 nm are represented by the shaded region and exist on the edge of ^7^F_0_→^5^L_6_ excitation lines of Eu^3+^. The strong red emission of Eu^3+^ balances the blue emission of Eu^2+^ and the colour changes from the red to yellow-white region. The emission almost reaches the white light at 410 nm.

**Figure 12:**
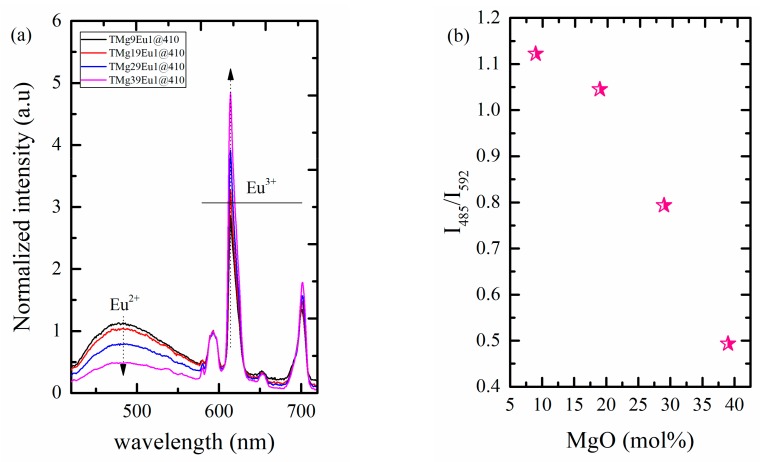
Graphic of (**a**) normalized emission spectra of the prepared glasses at λ_ex_ = 410 nm; (**b**) intensity ratio of Eu^2+^ emission (480 nm) to that of the pure magnetic transition of Eu^3+^ emission (592 nm).

As excitation with 410 nm gives the highest intensity of Eu^2+^ emission and the emission falls in the white region. This wavelength was used to record the emission spectra for all of the glasses and here we can follow how the host will affect the emission under certain excitations (see Figure 12a). The spectra show that the Eu^2+^ emission’s relative intensity compared to the normalized Eu^3+^ emission decreases with increasing MgO content. No shift was observed for the position (≈485 nm). A similar trend is observed with an excitation at 330 nm in Figure 8a, but at a higher wavelength (≈485 nm) compared to that reported for Duran glasses (≈450 nm) [35]. The ratio between the intensity of Eu^2+^ emission to the pure magnetic transition of Eu^3+^ (I_485_/I_592_) indicates that the decrease in the intensity of the Eu^2+^ emission is associated with increase in the intensity of the hypersensitive transition of Eu^3+^ that could propose an energy transfer from Eu^2+^ to Eu^3+^ (see Figure 12b).

**Figure 13:**
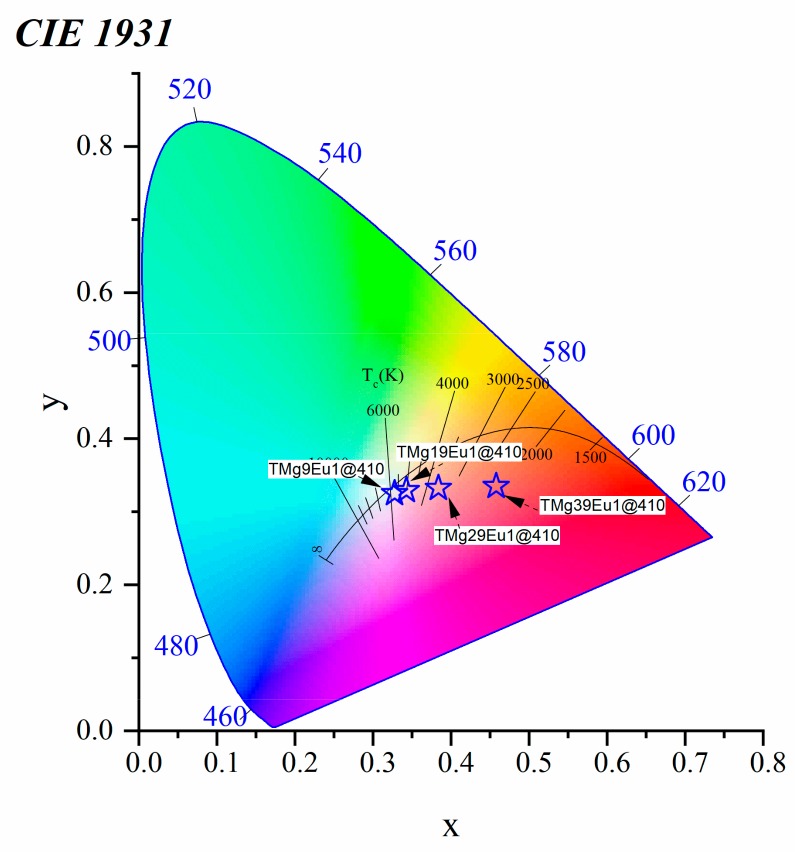
CIE 1931 chromaticity diagram for the prepared glasses at λ_ex_ = 410 nm.

The evolution of the intensity ratio between Eu^2+^ and Eu^3+^ allows us to tune the emitted color of our glasses as previously seen but this time using glass chemistry. The colour coordinates were computed from the spectra of Figure 12a, and are reported in Figure 13. From the Commission International de I’Eclairage (CIE) diagram, it can be noticed that the TMg9Eu1 is almost in the cool white region with its x, y coordinates (0.32, 0.32). As MgO increases, the colour coordinates of the emission under an excitation at 410 nm shift away from the white to the red-orange region.

## 4. Conclusions

In this study, we performed a thermal, structural, and spectroscopic analysis of binary magnesium tellurite glasses doped with 1 mol% of Eu_2_O_3_. The amorphous nature of the prepared glasses was proven using X-ray diffraction spectroscopy. The increase in the glass transition temperature for the doped glass compared to the undoped is associated with the high bond enthalpy of Eu_2_O_3_. Attenuated total reflection spectroscopy was used to study the structural change of the glass host. The transformation of the TeO_4_ trigonal bipyramid structural units to TeO_3_ trigonal pyramid is found to be higher in the doped glasses than the undoped, especially at high MgO content. The low phonon energy of Te-glass, and the decrease of the optical basicity of the glass host, all lead to more iconicity around Eu-ions. This leads to the increase of the intensity of the hypersensitive transition at 614 nm of Eu^3+^ and the decrease in the decay rate with increasing MgO content. Both Eu^2+^ and Eu^3+^ emissions were examined under different excitation wavelengths and the relative intensity of Eu^2+^ compared to Eu^3+^ was the highest with λ_exc_ = 410 nm, leading to a white emission. By increasing MgO content, Eu^2+^ emission intensity decreases. The color coordinates indicate that the emission of the TMg9Eu1 glass sample lies almost in the white region and shifts to the red-orange region with increasing MgO content. The results show that a good balance between Eu^2+^ and Eu^3+^ in tellurite glasses leads to white light emission.

## Figures and Tables

**Table 1:** Sample key, glass composition, glass transition temperature T_g_ (°C), optical basicity (^th) and decay rate (ms^−1^).

Sample Key	Composition	T_g_ ± 1.5 °C	^1^ T_g_* ± 1.5 °C	^th	Decay Rate (ms^−1^)
TMg9Eu1	90TeO_2_-9MgO-1Eu_2_O_3_	335	325	0.940	1.113
TMg19Eu1	80TeO_2_-19MgO-1Eu_2_O_3_	360	354	0.925	1.075
TMg29Eu1	70TeO_2_-29MgO-1Eu_2_O_3_	433	378	0.909	1.052
TMg39Eu1	60TeO_2_-39MgO-1Eu_2_O_3_	452	450	0.890	1.022

^1^ T_g_*: for the undoped corresponding glasses, see our previous work [18].

**Table 2:** The asymmetric ratio (R) of the prepared glasses and other reference values for other Eu-doped glasses.

Sample Key	R (^5^D_0_→^7^F_2_/^5^D_0_→^7^F_1_)	Reference
TMg9Eu1	3.77	Current study
TMg19Eu1	3.98	Current study
TMg29Eu1	4.55	Current study
TMg39Eu1	4.76	Current study
PKSAEu10	4.43	[38]
PbFBEu10	3.92	[39]
PKBFAEu10	4.69	[40]
LiPbAlBEu3	2.024	[16]
LiPbAlBEu7	2.468	[16]
ZnAlBiBEu0.1	1.951	[41]
ZnAlBiBEu2.5	2.78	[41]
BTeMgKEu1	4.12	[42]
BTeMgKEu2	4.24	[42]
BTeMgKEu3	4.14	[42]
PbFBAlWEu1	2.30	[43]
TeZnNaLiNbEu1	3.73	[44]
